# Integrating Active Learning Methodologies into Clinical Nutrition Education for Nursing Students: A Quasi-Experimental Study

**DOI:** 10.3390/nursrep15030077

**Published:** 2025-02-24

**Authors:** Stefano Mancin, Giovanni Cangelosi, Marco Sguanci, Sofia Matteucci, Emanuela Morenghi, Diego Lopane, Daniela Cattani, Simone Cosmai, Giulia Vinciguerra, Mauro Parozzi, Sara Morales Palomares, Beatrice Mazzoleni

**Affiliations:** 1 IRCCS Humanitas Research Hospital, Via Manzoni 56, 20089 Rozzano, Italy; stefano.mancin@humanitas.it (S.M.); sofia.matteucci@humanitas.it (S.M.); emanuela.morenghi@humanitas.it (E.M.); diego.lopane@humanitas.it (D.L.); daniela.cattani@humanitas.it (D.C.); giulia.vinciguerra@humanitas.it (G.V.); 2 Unit of Diabetology, Asur Marche–Area Vasta 4 Fermo, 63900 Fermo, Italy; giovanni.cangelosi@virgilio.it; 3 A.O. Polyclinic San Martino Hospital, 16132 Genova, Italy; 4 Department of Biomedical Sciences, Humanitas University, Via Rita Levi Montalcini 4, 20090 Pieve Emanuele, Milan, Italy; simone.cosmai@hunimed.eu (S.C.); beatrice.mazzoleni@hunimed.eu (B.M.); 5 School of Nursing, “San Paolo” Campus, Asst Santi Paolo e Carlo, University of Milan, 20129 Milan, Italy; 6 Department of Pharmacy, Health and Nutritional Sciences (DFSSN), University of Calabria, 87036 Rende, Italy; sara.morales@unical.it

**Keywords:** clinical nutrition, nursing students, active learning, education, information and communication technology, critical thinking

## Abstract

**Background**: Clinical nutrition is essential in nursing care, with nurses playing a key role in supporting patients’ dietary needs. **Aim**: To assess the impact of active learning methodologies on clinical nutrition education, focusing on knowledge retention among nursing students. **Methods**: Using a quasi-experimental research design, we enrolled 131 s-year nursing students. Both groups attended an eight-hour seminar on clinical nutrition, incorporating critical thinking. The experimental group had prior exposure to active learning and ICT in their first-year course, while the control group received traditional teaching. Knowledge was assessed using a validated questionnaire on basic and clinical nutrition. **Results**: The experimental group scored higher in both basic and clinical nutrition, indicating that active learning improves immediate learning and long-term retention. One year later, they retained significant knowledge, particularly regarding nutrient–disease relationships. **Conclusions**: Active learning, particularly critical thinking, enhances short- and long-term outcomes in clinical nutrition education. Future studies should refine assessment tools and explore further integration of active learning into nursing curricula.

## 1. Introduction

In recent decades, the field of clinical nutrition has gained increasing recognition as a fundamental element of patient-centered care, reflecting its essential role in the comprehensive management of health across diverse clinical settings [1,2]. Clinical nutrition can be defined as the branch of healthcare focused on assessing and optimizing nutritional intake to prevent and manage disease, supporting both recovery and long-term health outcomes [3].

Nutritional care, broadly defined as any intervention aimed at optimizing a patient’s dietary intake to improve health outcomes [3], is now considered a vital component of nursing practice [1]. The significance of nutrition in nursing extends beyond mere dietary recommendations; it is a critical factor in patient recovery, the management of chronic diseases, and the broader promotion of public health [4,5,6,7].

However, while the importance of nutritional care is widely acknowledged, its practical application in clinical settings remains complex. This complexity arises from multiple factors, including systemic pressures within healthcare environments, variations in nurses’ perceptions of their responsibilities related to nutritional care, and differing perspectives among healthcare professionals regarding the scope of nutrition-related duties within the nursing role [8,9]. These factors create barriers to the consistent and effective integration of nutritional care into everyday nursing practice, necessitating a closer examination of how such care is taught and implemented.

The necessity of comprehensive nutrition training for nurses cannot be understated, as it is crucial for ensuring timely and effective care that supports patient recovery, chronic disease management, and overall health promotion [9]. Despite this, current nursing education programs often fail to provide adequate training in clinical nutrition, resulting in a gap between the theoretical knowledge imparted during education and the practical skills required in professional practice [10,11]. This gap is particularly concerning given the assumption within many nursing curricula that nutritional care is an inherent competency of practicing nurses [12,13]. Such assumptions reveal a disconnect between university-level education and the realities of clinical practice, leaving many nursing graduates underprepared to meet the nutritional needs of their patients effectively [14].

Several studies have investigated the impact of active learning methodologies in nursing education, particularly regarding knowledge acquisition and retention in nutrition-related courses. Research suggests that e-learning platforms, interactive simulations, and blended learning approaches enhance students’ ability to retain complex nutritional concepts over time, fostering a more profound and applicable understanding of clinical nutrition in nursing practice [15,16].

These findings align with the objectives of our study, which evaluates the impact of active learning strategies on immediate and long-term learning outcomes in clinical nutrition education for nursing students. By fostering a more dynamic educational environment, these approaches can significantly enhance students’ understanding and retention of critical concepts in nutritional care, thereby better preparing them for clinical practice [17].

The adoption of e-learning and other digital materials in medical education has expanded substantially over the past decade. This expansion has been driven by technological innovations such as artificial intelligence (AI), deep learning (DL), and machine learning (ML), as well as by the increased reliance on digital education methods during the COVID-19 pandemic [18]. Numerous studies have demonstrated that active learning strategies are superior to traditional didactic approaches, particularly in enhancing conceptual understanding, critical thinking, and problem-solving skills that are essential for effective nursing practice [19,20,21]. Although the field of electronic content development in education is still relatively new, it is rapidly growing, with significant potential for further development in the coming years [18].

Nevertheless, there remains a notable lack of research focused specifically on the outcomes and knowledge retention associated with nutritional education in nursing. This is especially true when comparing active learning methodologies with more traditional approaches. The existing gap in the literature underscores the urgent need for empirical research to evaluate the effectiveness of these new teaching methodologies in enhancing the nutritional education of nursing students and ensuring long-term knowledge retention [19].

The objective of this study was to compare the effectiveness of two different teaching methodologies applied to clinical nutrition education in two homogeneous groups of students. Both groups participated in a clinical nutrition course based on the Critical Thinking methodology; however, one group had previously attended a foundational nutrition course using an active learning methodology with an expert instructor, while the other group had taken part in a traditional face-to-face course using conventional teaching methods. A secondary objective was to evaluate knowledge retention trends in the experimental group by comparing the level of knowledge acquired during the previous course with that retained one year later.

## 2. Materials and Methods

### 2.1. Study Design

This is a quasi-experimental research study comparing two groups of second-year nursing students who participated in an eight-hour seminar on Clinical Nutrition. This study was conducted in accordance with the Strengthening the Reporting of Observational Studies in Epidemiology (STROBE) guidelines [22].

The experimental group (Group A), enrolled in the first-year nutrition course at one campus of the university, completed the course utilizing active learning methodologies, including Information and Communication Technologies (ICT) tools, case-based learning, and Audience Response Systems (ARS) to promote interactive engagement. The control group (Group B), from a different campus of the same university, attended a traditional lecture-based course on the same nutritional topics without these interactive methodologies. The study aimed to assess knowledge retention after one year of exposure to different teaching methods.

### 2.2. Participants, Criteria, and Context

A total of 131 students participated in the study. The sample was a voluntary, convenience sample, and recruitment occurred through the university’s Learning Management System (LMS), where an electronic link to the survey was provided. All participants gave informed consent for the use of their data in the study.

The inclusion criteria were students enrolled in the second year of the Bachelor’s Degree in Nursing at the Faculty of Medicine and Surgery of a university in Northern Italy. Exclusion criteria included students who had repeated the second year or those in Group A who had not completed the nutrition course using active learning methodologies.

Students self-selected into either the experimental group (Group A), members of which attended the course utilizing active learning methodologies, or the control group (Group B), members of which attended the traditional lecture-based course. The different sample sizes in the two groups resulted from this self-selection process.

### 2.3. Educational Intervention and Course Design

In the second year, students attended a specific seminar on clinical nutrition, focusing on its application to chronic diseases such as cardiovascular, renal, and metabolic conditions, as well as in the management of surgical patients. The course granted one university credit (ECTS) and was delivered in a full-day session. Additionally, during their first year of study, all students had completed a module worth seven ECTS, spread over 17 weeks, including 0.5 ECTS focused on clinical nutrition and nursing care based on Gordon’s functional health patterns [23]. The educational intervention in this study involved two main components, which were implemented in both the first and second years of the nursing program:

(a) Experimental Group (Group A): This group participated in a first-year nutrition course that incorporated active learning methodologies. The course included video-based content, interactive quizzes, and ARS such as Wooclap (https://www.wooclap.com), enabling real-time participation [24]. The integration of ICT tools, as demonstrated in previous research [25,26], facilitated enhanced student engagement, deeper understanding, and increased participation. The ARS allowed students to interact with the content through various activities, including multiple-choice questions, polls, and open-ended questions, while also providing the option to signal difficulties in real time.
(b) Control Group (Group B): This group participated in a first-year nutrition course and attended a traditional lecture-based course in their first year, which did not include the use of active learning tools or digital engagement methods.

**Figure 1:**
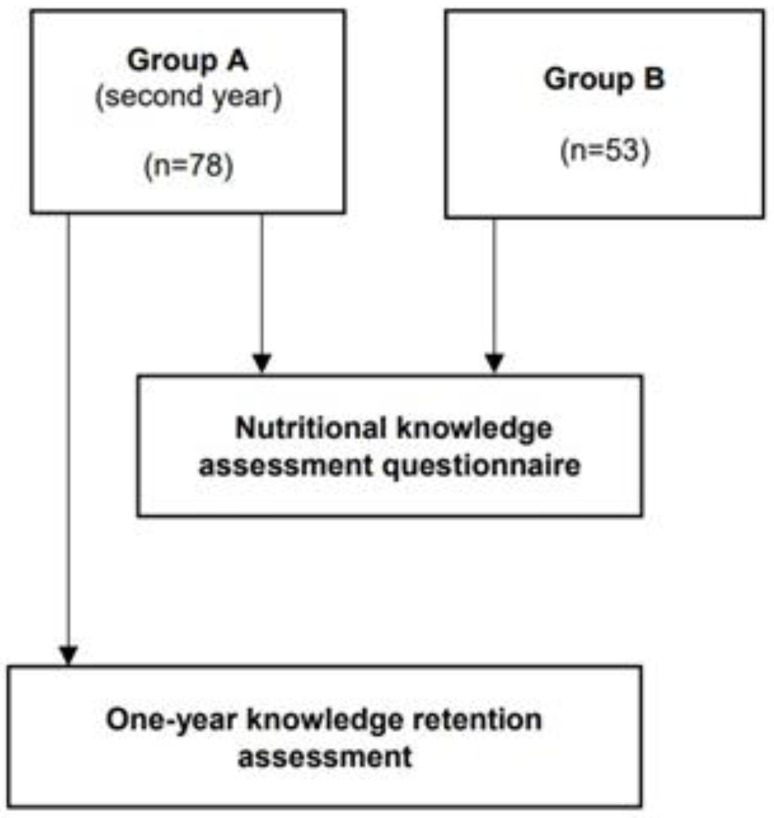
Sample and Study Procedures—Legend. Group A = Experimental group; Group B = Control group.

Both groups participated in the same second-year seminar on clinical nutrition, which focused on the application of clinical nutrition to chronic diseases such as cardiovascular, renal, and metabolic conditions, as well as the management of surgical patients. This seminar, delivered as a full-day session, granted one ECTS credit. The seminar employed a traditional face-to-face instructional approach, enhanced by the integration of critical thinking methodologies [26] (Figure 1).

Critical thinking, in the context of education, involves questioning, analyzing, interpreting, evaluating, and making judgments about information, whether derived from reading, listening, writing, or speaking. In academic settings, critical thinking is often promoted through discussions and debates during lectures [21]. The effectiveness of critical thinking as a teaching method is grounded in several key principles [18], including defining the scope and context of the subject matter, critically evaluating information sources, analyzing arguments, considering diverse viewpoints, and synthesizing or formulating one’s own arguments.

In this study, critical thinking was embedded within the traditional face-to-face teaching framework and further reinforced through methods that fostered inductive reasoning [27]. As supported by previous research [17,18,28], the critical thinking methodology was facilitated by carefully designed questions aimed at stimulating analysis and reasoning, encouraging students to engage critically with the content during the seminar.

### 2.4. Measurement

In this study, the level of knowledge regarding basic clinical nutrition concepts was assessed using a previously validated Italian questionnaire [29]. The original questionnaire was developed by Moynihan et al. [30] and was an abbreviated version of a questionnaire created by Parmenter and Wardle [31].

The tool has demonstrated adequate psychometric properties for use in clinical research. It consists of 15 questions: the first section (questions 1–10) assesses general nutritional knowledge, while the second section (questions 11a–11e) focuses on more specific knowledge related to nutrition in the context of diseases caused by poor dietary choices (clinical nutrition).

Correct answers are scored with 1 point, and incorrect responses receive 2 points. For multiple-choice questions (questions 4 and 6), a score of 0.1 is given for each correct answer and 0.2 for each incorrect one. The total scoring ranges from a minimum of 15 points (representing the best possible score) to a maximum of 30 points (indicating the worst score).

Additionally, questions 11a through 11e allow for qualitative analysis, which provides a more detailed evaluation of participants’ knowledge of specific topics. Although the qualitative data do not influence the overall score, they are used for descriptive analysis to offer a more nuanced understanding of the participants’ nutritional knowledge.

Students who voluntarily enrolled in the study completed the nutritional knowledge questionnaire within one to three days after the Clinical Nutrition seminar. The collected data were then used to compare the questionnaire results between Group A and Group B. Additionally, knowledge retention was assessed for Group A only, as these students voluntarily completed the same questionnaire at the end of the first-year nutrition course. This allowed for an evaluation of the long-term effectiveness of the active learning methods by comparing scores at two time points. However, due to the anonymous and voluntary nature of participation, only the overall trend in knowledge retention could be analyzed. The aggregated data made it impossible to assess retention at an individual level.

### 2.5. Data Analysis

As the study had no pre-specified difference to be demonstrated, the objective was exploratory and participation was on a voluntary basis, no sample size calculation was performed a priori, and the study enrolled all of the students who agreed to participate. The final sample size allowed us to determine significance, with a 5% alpha error, 80% power, and an effect size of 0.5 [32]. Data were described as number and percentage, if categorical, or mean and standard deviation, if numerical. Differences between groups were explored with the chi square test, with Fisher correction if necessary, for categorical variables, or with Student’s *t* test or the Mann–Whitney test for numerical variables, according to their distribution. Adherence to Gaussian distribution was verified with the Shapiro–Wilk test. The association between total score and the intervention was explored with linear regression analysis, and the results were presented with adjustment for age and sex. The significance threshold was set at 0.05. All analyses were performed with Stata version 18.

### 2.6. Ethical Considerations

Approval to conduct the study was obtained from the Humanitas University ethics committee in Pieve Emanuele, Italy, under approval number CLI_RIC_02/2024. All participants provided written informed consent before taking part in the study.

## 3. Results

### 3.1. Sample Characteristics

The study involved a total sample of 131 s-year nursing students, divided into two groups: Group A (n = 78) and Group B (n = 53). The two groups were comparable in terms of gender (males 19 (24.4%) in Group A vs. 7 (13.2%) in Group B, χ^2^ = 2.467, *p* = 0.116) and age (23.8 ± 4.6 years in Group A and 23.2 ± 4.2 years in Group B, z = −0.724, *p* = 0.469)

### 3.2. Nutritional Knowledge

**Figure 2:**
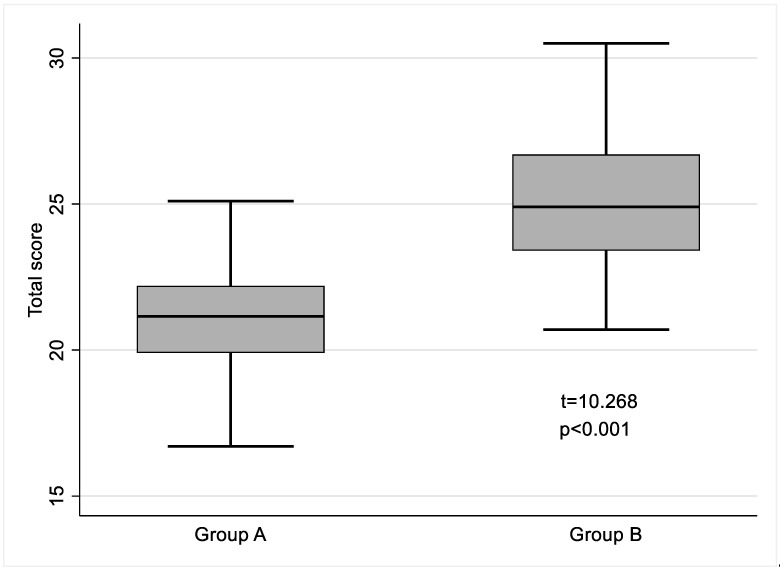
Nutritional Knowledge—Legend. Group A = Experimental group; Group B = Control group.

The questionnaire results showed differences between the groups with a response score of 21.3 ± 1.7 for Group A and 24.9 ± 2.3 for Group B (t = 10.268, *p* < 0.001) (Figure 2).

For questions 1–10, which assessed nutritional knowledge, the mean scores were 16.0 ± 1.5 for Group A and 18.7 ± 2.0 for Group B (z = 7.139, *p* < 0.001).

We assessed whether the intervention independently influenced the scores for questions 1–10. The results, adjusted for age and sex, indicated that the intervention group had significantly better scores compared to the control group (coefficient: −2.815, *p* < 0.001), suggesting a direct effect of the active learning methodology on reducing the performance gap in basic nutritional knowledge, independent of age and sex. Neither age (*p* = 0.937) nor gender (*p* = 0.266) had a significant effect on scores (Table 1).

### 3.3. Clinical Nutritional Knowledge

The results showed that, for the questions in domain 11, Group A had a total score of 5.31 ± 0.79 with a level of “Yes” responses regarding the impact of nutrients on different pathologies of 93.8% ± 15.9%, compared to Group B, which had a score of 6.15 ± 1.36 and a response percentage of 77.0% ± 27.3% (z = −4.242, *p* < 0.001).

A more detailed analysis was conducted on students’ responses related to fiber-related health issues (11a), fruit and vegetable-related health issues (11b), fat-related health issues (11c), sugar-related health issues (11d), and salt-related health issues (11e). In the analysis of dietary knowledge between Group A and Group B, significant differences emerged across several areas (Table 2).

The analysis of fiber-related issues revealed greater awareness in Group A, with a significantly higher proportion of correct responses regarding conditions such as constipation. Group B, while aware of fiber’s importance, showed a significantly lower response rate.

Regarding fruit and vegetable consumption, Group A more frequently identified the consequences of nutritional deficiencies and reduced immune function compared to Group B. Both groups demonstrated solid awareness of the health risks associated with dietary fats, with no significant differences. However, in terms of excessive sugar consumption, Group A showed a markedly better understanding than Group B. Finally, with respect to salt consumption, Group A exhibited greater awareness of its harmful effects compared to Group B, further confirming the trend of superior dietary knowledge in Group A (Table 2).

### 3.4. Nutritional Knowledge Retention

An additional analysis was performed for Group A to evaluate the retention of knowledge one year after completing the first basic clinical nutrition course, which employed active teaching methodologies (Table 3). A total of 63 participants (males: n = 16, 25.40%; mean age: 22.4 ± 5.4 years) completed the questionnaire during the first year.

During the first year, Group A participated in a course focused on both basic and clinical nutrition, structured around the Gordon nursing model, achieving a total score of 22.0 ± 3.0. This was compared to a score of 21.3 ± 1.7 during the second-year assessment, which is the focus of this study. Despite the slight decrease, the results indicate substantial retention of nutritional knowledge over a 12-month period, with no statistically significant difference between the two assessments (t = 1.903, *p* = 0.296).

Further analysis of the experimental group’s knowledge retention after one year revealed an overall improvement in dietary knowledge. The total score for nutrient-related diseases increased significantly from the first to the second year, rising from 85.4% ± 21.9% to 93.8% ± 15.9% (z = 2.927, *p* = 0.003). When examining specific knowledge areas (questions 11a–e), individuals in all categories either maintained or improved their knowledge levels compared to the first year.

## 4. Discussion

This study evaluated the impact of active learning methodologies on clinical nutrition education among a sample of second-year nursing students, focusing specifically on knowledge retention between the first and second years of the program. The findings highlight the effectiveness of active teaching methodologies in enhancing students’ nutritional knowledge, corroborating results previously observed in the international literature [27,28].

The importance of nutritional knowledge in nursing care has been widely studied and is recognized as critical for managing various diseases. Specifically, several studies emphasize how proper nutritional management can significantly improve outcomes in patients with various chronic conditions [33,34]. According to previous studies [35], adequately trained nurses can deliver effective nutritional interventions, reducing the risk of complications and promoting healthier lifestyles in patients. Similarly, in the context of cardiovascular diseases, nutritional education plays a pivotal role in both prevention and management [36]. A previous study [37] highlights how nurses with strong nutritional knowledge can effectively reduce cardiovascular risk factors through targeted dietary interventions and patient education, thereby contributing to better patient outcomes and reducing the strain on healthcare systems. Furthermore, in the management of other conditions such as chronic diseases like diabetes, nutritional assessment and intervention are crucial not only for identifying malnutrition but also for preventing complications and disease progression and promoting prevention from a lifestyle medicine perspective [38]. This highlights the importance of specific nutritional training for nursing students, who will later work in these fields and play a key role in supporting patients throughout their therapeutic journeys [27].

The analysis of scores related to basic nutritional knowledge revealed significant differences between the two groups, despite both having received face-to-face instruction integrated with active learning methodologies centered on critical thinking. Group A, members of which had previously participated in a course during the first year utilizing active learning methodologies based on critical thinking and supplemented with ICT, achieved a lower mean score compared to Group B, members of which had received traditional face-to-face training. Since a lower score corresponds to better performance, this suggests that students previously exposed to active methodologies assimilated and retained basic nutritional knowledge more effectively.

Critical thinking was the key element in the active learning methodologies employed in this study. Unlike passive knowledge transmission, this methodology encourages students to develop skills in analyzing, evaluating, and synthesizing information [39]. During lessons, students were prompted to question sources, evaluate arguments, and form their own judgments. The seminar structure fostered the use of targeted questions designed to stimulate inductive reasoning and critical reflection. This approach enabled a deeper understanding of the concepts, moving beyond mere memorization, as confirmed by the international literature [40].

The significance of critical thinking is particularly evident in nursing sciences, where the ability to assess and make informed clinical decisions is essential. Several studies [41,42] have demonstrated that integrating this educational approach in health sciences improves active learning and the practical application of knowledge, promoting student autonomy and critical thinking. In our study, the use of this methodology contributed to improved student performance in acquiring and retaining nutritional knowledge, proving more effective than traditional teaching methods. Regarding Group A, in addition to the integration of critical thinking, ICT was used as a supplementary tool during first-year training. Although it was not a direct component of the active learning methodologies in the second year, the use of ICT resources in the first year provided additional support to students’ learning, allowing quicker and more interactive access to content. The use of educational videos and other digital tools has been shown to enhance skill acquisition and improve student engagement, as evidenced by several studies [43].

In terms of specific knowledge in clinical nutrition, Group A once again demonstrated better scores compared to Group B, particularly in questions related to the correlation between nutrients and specific pathologies. This reflects an ability to apply nutritional knowledge to clinical practice, indicative of deeper learning. Supporting this, the effectiveness of active methodologies in clinical nutrition education is well documented in the literature. Several studies [44,45] emphasize that direct interaction with clinical content through simulations and case discussions allows students to connect theory and practice, significantly enhancing their ability to apply knowledge in real-world settings. This type of learning also increases students’ confidence in handling complex clinical scenarios, such as managing patients with chronic nutrition-related diseases.

One particularly interesting finding from the study is the retention of knowledge one year after the educational intervention. Group A retained a significant portion of the competencies acquired, especially in questions on the relationships between nutrients and diseases. This suggests that active learning methodologies not only improve immediate learning but also facilitate long-term knowledge retention. This phenomenon is supported by studies such as Minnick et al. [19], which show that active learning, compared to traditional methods, leads to greater knowledge retention over time. This may be due to the fact that active methodologies require students to continually process and reprocess information, thereby reinforcing long-term memory.

### Study Limitations

This study presents several limitations that must be acknowledged. Firstly, the tool used to assess knowledge, although effective in evaluating both basic and clinical nutrition concepts and designed for healthcare professionals, may not fully align with the specific educational objectives of nursing students. Future research should consider replacing it with a questionnaire specifically tailored to the nursing curriculum to ensure a more accurate reflection of the competencies taught. Another limitation lies in the lack of available data regarding Group B’s first-year course, making it difficult to comprehensively compare the two groups. Additionally, although knowledge retention was assessed within the same sample, the findings can only suggest a trend, as the questionnaire was anonymous and voluntary, potentially resulting in variability in the student population between the two assessment points. This variability may have impacted the consistency of the results. One potential limitation of this study is the comparison between groups with different first-year learning experiences, which may introduce a source of bias. While both groups received the same second-year clinical nutrition seminar, variations in teaching methodologies during their first year could have influenced their learning process and retention. Additionally, differences in individual learning abilities and cohort characteristics may have contributed to performance disparities.

Future research should consider a controlled experimental design, ensuring that both groups undergo the same first-year educational experience, to further validate the impact of active learning methodologies on long-term knowledge retention.

## 5. Conclusions

This study highlights the positive impact of active learning methodologies, particularly those centered on critical thinking, on the acquisition and retention of clinical nutritional knowledge among second-year nursing students. The findings indicate that integrating these methodologies into nursing education enhances not only immediate knowledge acquisition but also long-term retention, as evidenced by the improved performance of the experimental group in both basic and clinical nutrition assessments. The critical thinking approach, combined with interactive tools like ICT, proved essential in helping students actively engage with course content, allowing them to move beyond rote memorization and apply their knowledge in clinical practice.

Future research should aim to develop more tailored assessment tools for nursing students and ensure a more comprehensive analysis of educational interventions. Nevertheless, the findings provide valuable insights into the benefits of active learning in nursing education, particularly in the field of clinical nutrition, and suggest its potential to enhance academic performance and long-term knowledge retention, ultimately preparing nursing students for effective clinical practice.

## Figures and Tables

**Table 1:** Effect of the intervention on nutritional knowledge scores.

Variables	Coefficient (95%CI)	*p*
Group A vs. Group B	−2.815 (−3.425 to −2.206)	<0.001
Age	−0.003 (−0.071 to 0.065)	0.937
Gender (m)	0.430 (−0.332 to 1.192)	0.266
Cons	18.739 (17.111 to 20.366)	<0.001

Legend: CI = 95% confidence interval; Group A = Experimental group; Group B = Control group; m = Male; Cons = Intercept of the regression model; *p* = *p*-value.

**Table 2:** Specific dietary knowledge.

	Group A	Group B	χ2	p (A vs. B)
n	78	53		
Question 11a	73 (93.6%)	37 (69.8%)	13.256	<0.001
Question 11b	69 (88.5%)	41 (77.4%)	2.890	0.097
Question 11c	73 (93.6%)	44 (83.0%)	3.695	0.082
Question 11d	76 (97.4%)	42 (79.2%)	11.682	0.002
Question 11e	75 (96.2%)	40 (75.5%)	12.590	0.001

Legend: Group A = Experimental group; Group B = Control group. Questions on health issues related to fiber (11a), fruits and vegetables (11b), fats (11c), sugars (11d), and salt (11e).

**Table 3:** Knowledge retention.

Knowledge	Group A (First Year)	Group A (Second Year)	Test Value	*p*
n	63	78		
Total score	22.0 ± 3.0	21.3 ± 1.7	1.903 *	0.059
Nutritional knowledge	16.3 ± 2.6	16.0 ± 1.5	1.047 **	0.296
Clinical nutritional knowledge	5.7 ± 1.1	5.3 ± 0.8	2.927 **	0.003
Nutrient-related diseases (total score) (%)	85.4 ± 21.9	93.8 ± 15.9	2.927 **	0.003
Fiber-related health issues (%)	43 (68.3%)	73 (93.6%)	15.336 ^#^	<0.001
Fruit and vegetable-related health issues (%)	53 (84.1%)	69 (88.5%)	0.562 ^#^	0.469
Fat-related health issues (%)	59 (93.7%)	73 (93.6%)	0.000 ^#^	1.000
Sugar-related health issues (%)	60 (95.2%)	76 (97.4%)	0.492 ^#^	0.656
Salt-related health issues (%)	54 (85.7%)	75 (96.2%)	4.878 ^#^	0.035

Legend. Group A = Experimental group. * *t* test, ** Mann–Whitney test, and ^#^ χ^2^ test.

## Data Availability

The data used in this study can be requested from the corresponding author of the manuscript.

## Public Involvement Statement

No public involvement in any aspect of this research.

## Guidelines and Standards Statement

This manuscript was drafted based on the Strengthening the Reporting of Observational Studies in Epidemiology (STROBE) guidelines [22].
